# Redo Surgical Aortic Valve Replacement versus Valve-In-Valve Transcatheter Aortic Valve Implantation: A Systematic Review and Reconstructed Time-To-Event Meta-Analysis

**DOI:** 10.3390/jcm12020541

**Published:** 2023-01-09

**Authors:** Francesco Formica, Alan Gallingani, Domenico Tuttolomondo, Daniel Hernandez-Vaquero, Stefano D’Alessandro, Claudia Pattuzzi, Mevlüt Çelik, Gurmeet Singh, Evelina Ceccato, Giampaolo Niccoli, Roberto Lorusso, Francesco Nicolini

**Affiliations:** 1 Department of Medicine and Surgery, University of Parma, 43124 Parma, Italy; 2 Cardiac Surgery Clinic, University Hospital of Parma, Via Gramsci 14, 43125 Parma, Italy; 3 Cardiology Unit, University Hospital of Parma, Via Gramsci 14, 43125 Parma, Italy; 4 Cardiac Surgery Department, Hospital Universitario Central de Asturias, 33004 Oviedo, Spain; 5 Cardiac Surgery Unit, San Giovanni Bosco Hospital, 10144 Turin, Italy; 6 Department of Cardiothoracic Surgery, Erasmus University Medical Center, 3062 Rotterdam, The Netherlands; 7 Division of Cardiac Surgery, Department of Critical Care Medicine, Mazankowski Alberta Heart Institute, University of Alberta, Edmonton, AB 11220, Canada; 8 Medical Library, University of Parma, 43124 Parma, Italy; 9 Cardiovascular Research Institute Maastricht (CARIM), 6200 Maastricht, The Netherlands

**Keywords:** biological prosthesis valve degeneration, redo surgical aortic valve replacement, valve-in-valve transcatheter aortic valve implantation, meta-analysis, long-term outcome

## Abstract

Objective. Valve-in-valve transcatheter aortic valve implantation (ViV-TAVI) has emerged as a useful alternative intervention to redo-surgical aortic valve replacement (Redo-SVAR) for the treatment of degenerated bioprosthesis valve. However, there is no robust evidence about the long-term outcome of both treatments. The aim of this meta-analysis was to analyze the long-term outcomes of Redo-SVAR versus ViV-TAVI by reconstructing the time-to-event data. Methods. The search strategy consisted of a comprehensive review of relevant studies published between 1 January 2000 and 30 September 2022 in three electronic databases, PubMed, Cochrane Central Register of Controlled Trials (CENTRAL) and EMBASE. Relevant studies were retrieved for the analysis. The primary endpoint was the long-term mortality for all death. The comparisons were made by the Cox regression model and by landmark analysis and a fully parametric model. A random-effect method was applied to perform the meta-analysis. Results. Twelve studies fulfilled the eligibility criteria and were included in the final analysis. A total of 3547 patients were included. Redo-SAVR group included 1783 patients, and ViV-TAVI included 1764 subjects. Redo-SAVR showed a higher incidence of all-cause mortality within 30-days [Hazard ratio (HR) 2.12; 95% CI = 1.49–3.03; *p* < 0.0001)], whereas no difference was observed between 30 days and 1 year (HR = 1.03; 95% CI = 0.78–1.33; *p* = 0.92). From one year, Redo-SAVR showed a longer benefit (HR = 0.52; 95% CI = 0.40–0.67; *p* < 0.0001). These results were confirmed for cardiovascular death (HR = 2.04; 95% CI = 1.29–3.22; *p* = 0.001 within one month from intervention; HR = 0.35; 95% CI = 0.18–0.71; *p* = 0.003 at 4-years follow-up). Conclusions. Although the long-term outcomes seem similar between Redo-SAVR and ViV-TAVI at a five-year follow-up, ViV-TAVI shows significative lower mortality within 30 days. This advantage disappeared between 30 days and 1 year and reversed in favor of redo-SAVR 1 year after the intervention.

## 1. Introduction

The most recent guidelines on heart valve disease [1] recommend a selection of a biological prosthesis (BP) valve for patients with aortic stenosis (AS) older than 60 years, and the use of transcatheter aortic valve implantation (TAVI) is strongly recommended in patients older than 75 years or in high-risk patients. A major advantage of the BP valve is the avoidance of lifelong anticoagulation therapy. This advantage has led over time to a significant increase in the use of BP valves in young patients [2,3]. Despite the long durability of BP valves, these valves are associated with a high degeneration rate within 15 years after the intervention and may necessitate re-operation even at an earlier post-implant time in young patients [4,5]. Repeat surgical aortic valve replacement (Redo-SAVR) is the gold standard for the treatment of degenerated aortic BP valves, but it is burdened with significant surgical risk, especially in those patients with patent coronary artery grafts. The non-inferiority of TAVI procedures [6,7] has led to the expansion of the use of this less invasive approach for a valve-in-valve TAVI (ViV-TAVI) [8]. However, randomized trials comparing the two interventional strategies (Redo-SAVR vs. ViV-TAVI) are currently lacking, and most data and results come from observational studies [9,10]. Recently, some meta-analyses [11,12,13,14] have reported interesting data regarding the comparison between Redo-SAVR and ViV-TAVI in terms of short- and long-term outcomes, but with no evidence of time-to-event analysis. Accordingly, we performed a comprehensive systematic review and meta-analysis with the aim of comparing long-term survival by reconstructing the time-to-event data between Redo-SAVR and ViV-TAVI and further focusing on the potential time-varying effect and modeling their hazard ratio over time.

## 2. Materials and Methods

This meta-analysis is exempted from Ethical Committee (EC) evaluation and approval because the data were extracted from published articles in which the investigators had obtained approval from the local ECs. The meta-analysis adhered to the Preferred Reporting Items for Systematic Reviews and Meta-Analyses (PRISMA) guidelines [15] and Meta-analysis of Observational Studies in Epidemiology (MOOSE) guidelines [16]. The study protocol was registered and published online in PROSPERO (The International Prospective Register of Systematic Reviews; ID: CRD42022313070).

### 2.1. Eligibility Criteria, Search Strategy and Selection Process

We followed the PICOS format (Population; Intervention; Comparison; Outcomes; Studies) for the eligibility studies. Population: patients with degenerated bioprosthetic aortic valve; Intervention: patients who underwent Redo-SAVR; Comparison: patients who received a ViV-TAVI; Outcomes: studies that reported at least 1-year follow-up and a graphed Kaplan–Meier (KM) curves of survival and freedom from cardiovascular (CV) death; Studies: observational studies and randomized control trials. The search strategy consisted of a comprehensive review of relevant studies published between 1 January 2000 and 30 September 2022 in three electronic databases, PubMed, Cochrane Central Register of Controlled Trials (CENTRAL) and EMBASE. Moreover, the references list of relevant articles was used to complete the search. The following search terms were used alone and in combination: “redo surgical aortic valve replacement”, “redo-SAVR”, “reoperation”, “reoperative”, “valve-in-valve”, “ViV-TAVI”, “ViV-TAVR”, “transcatheter”. A medical librarian (EC) implemented the search through the abovementioned electronic databases. The search algorithm is reported in Supplementary Material, Table S1. The meta-analysis was conducted based on the PRISMA checklist (Supplemental Material checklist Table S1) and the studies have been selected following these steps: (1) identification of titles and abstracts of records in electronic databases; (2) removal of duplicates; (3) screening and selection of abstracts; (4) retrieval of full text-papers and assessment for eligibility through full-text papers; and (5) inclusion of the records in final analysis. Studies written in languages other than English, case reports, letters to Editor, editorials, meta-analyses and review articles were excluded. Four authors (SDA, AG, DT, CP) independently scanned and reviewed titles and abstracts, and disagreement was resolved with a senior author (FF).

### 2.2. Data Extraction and Collection

Four authors (SDA, AG, DT, CP) independently performed data extraction from text and appendix, which were then collected in a standard table sheet database (Microsoft Office Excel 2016, Microsoft, Redmond, WA, USA). The included studies were listed by first author, country, study design, study period and year of publication. The following patient baseline variables were extracted and collected: male gender, mean age, previous acute myocardial infarction (AMI), previous percutaneous coronary intervention (PCI), previous coronary artery bypass grafting (CABG), mean ejection fraction (EF), persistent/permanent atrial fibrillation (AF), hypertension, diabetes, cerebrovascular events (CVE), renal failure, dialysis, chronic obstructive pulmonary disease (COPD), peripheral vascular disease (PVD), history of smoking, dyslipidemia. The following peri and postoperative variables were also extracted and collected: new-onset AF, acute kidney injury (AKI), AKI requiring dialysis, permanent pacemaker (PM) implantation, CVE, new AMI, major bleeding, need for re-thoracotomy, blood transfusion, major vascular complications, prosthetic valve leakage, valvular mean gradient, patient-prosthesis mismatch (PPM), mean length of hospital stay (days), early mortality (30 days/in-hospital death), mean follow-up and longest follow-up. 

### 2.3. Risk of Bias Assessment

Two authors (DT, FF) estimated the risk of bias assessment using the Newcastle–Ottawa Scale (NOS) for non-randomized observational studies to assess the level of quality of included studies (Supplementary material, Table S2). 

### 2.4. Primary and Secondary Endpoints

The primary endpoint was the incidence of all-cause mortality at 5 years of follow-up. The secondary endpoint was the incidence of CV death at follow-up.

### 2.5. Statistical Analysis

Continuous variables were reported as mean ± standard deviation. Categorical variables were reported as numbers and percentages. Median and interquartile ranges were converted into mean and standard deviations using a validated formula [17]. We employed a dedicated software [GetData Graph Digitizer version 2.5.3] to digitize K-M curves. As extraction method, we opted for the curve approach, extracting ordinate (y) and abscissa (x) values from the K-M curves to calculate the hazard ratio (HR) for each time interval for which the number at risk is reported. This method allows reconstruction of time-to-event data at the individual level, and it overcomes the limitation of nonproportional hazard risks that are frequently reported in clinical trials [18,19]. 

The HR and the corresponding 95% CI were calculated by analyzing the time-to-event outcomes according to the methods proposed by Tierney et al. [20]. Then, we reconstructed the original database of each article using the method described by Wei et al. [19]. Quality assessment of reconstructed time-to-event data was performed by comparing the derived Kaplan–Meier curves visually with the original ones. Then, we created a single database by merging all the reconstructed databases to recalculate the aggregated survival curves and life tables. Treatment group comparisons were made by use of a Cox regression linear frailty model, with study as a random effect to account for the heterogeneity among the studies.

Proportionality of hazards was assessed by plotting log-minus-log survival curves, the scaled Schoenfeld residuals and predicted versus observed survival functions. We planned to perform a landmark analysis for each endpoint in the case of the violation of the test, indicated as a *p* < 0.05, The time-varying HRs of endpoints were modeled with the fully parametric generalized survival models, according to the Royston–Parmar models and using a restricted cubic-splines.

To assess the robustness of the synthesized results, a sensitivity analysis was conducted analyzing the primary endpoint in a subgroup of studies with propensity score matched (PSM) comparison, as this statistical method allows to control for the bias of confounder variable in non-randomized control trials. HR equivalence was set at 1, being HR < 1 on favor of redo-SAVR and HR > 1 on favor of ViV-TAVI. A two-tailed *p*-value < 0.05 indicated statistical significance. Statistical analyses were computed with Stata/SE version 16.1 (Stata Corp, College Station, TX, USA).

## 3. Results

**Figure 1:**
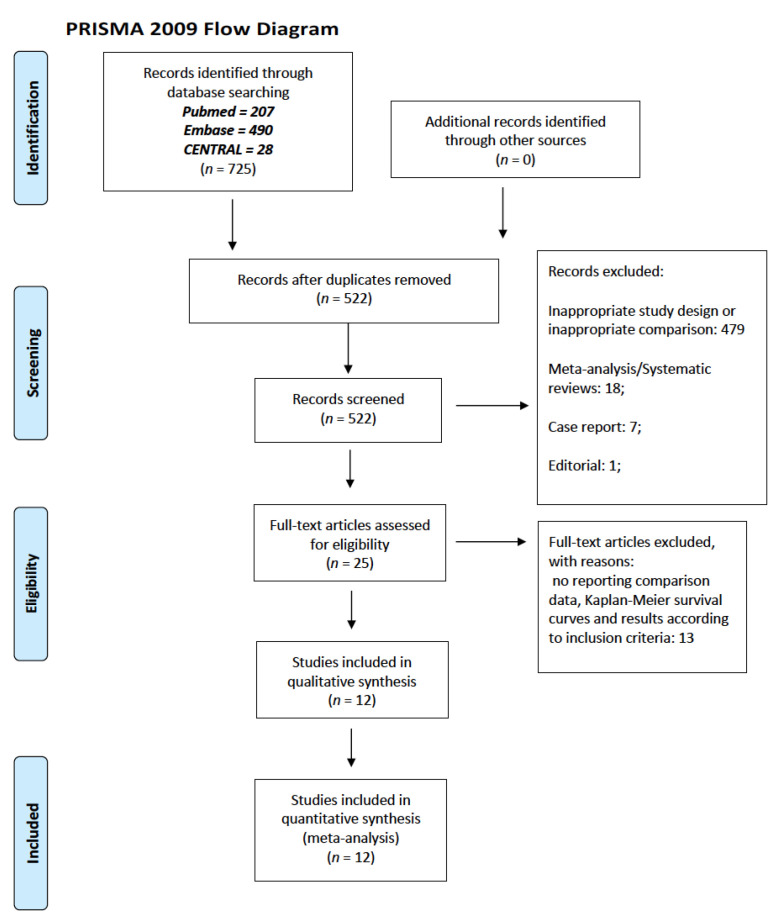
PRISMA flow chart.

A total of 725 records were identified, and 25 studies were considered relevant and retrieved. Among them, 12 studies fulfilled the eligibility criteria and were included in the final analysis [21,22,23,24,25,26,27,28,29,30,31,32]. The PRISMA Flow Chart of the study selection process is shown in Figure 1. 

All studies were non-randomized observational studies, and six of them [21,23,24,25,26,28] reported a comparison of matched populations. No randomized control trials were found, and no studies were withdrawn due to overlap with other studies.

A total of 3547 patients were included in the meta-analysis. Redo-SAVR group included 1783 patients, and ViV-TAVI included 1764 subjects. The weighted frequency of males was 53%. The study frame of included studies ranged between 2002 and 2019. Redo-SAVR patients were mostly younger (weighted mean, 66.9 years vs. 69.3 years, respectively), with lower frequencies of diabetes, stroke, COPD, PVD, AF and history of CABG in comparison with ViV-TAVI patients. The most frequent surgical approach for TAVI was transfemoral access. The baseline and postoperative variables of included studies are listed in Supplemental Tables S3 and S4, respectively.

### 3.1. Primary Endpoint: Long-Term Mortality

All included studies reported the long-term survival and the Kaplan–Meier curves comparison between Redo-SAVR and ViV-TAVI. The reported mean follow-up ranged between 1 year [23,29] and 3.2 years [28], with a weighted mean follow-up of 1.54 years. The longest follow-up was 10.4 years [28] and seven studies [23,24,25,26,28,29,30] reported a follow-up longer than 1 year.

**Figure 2:**
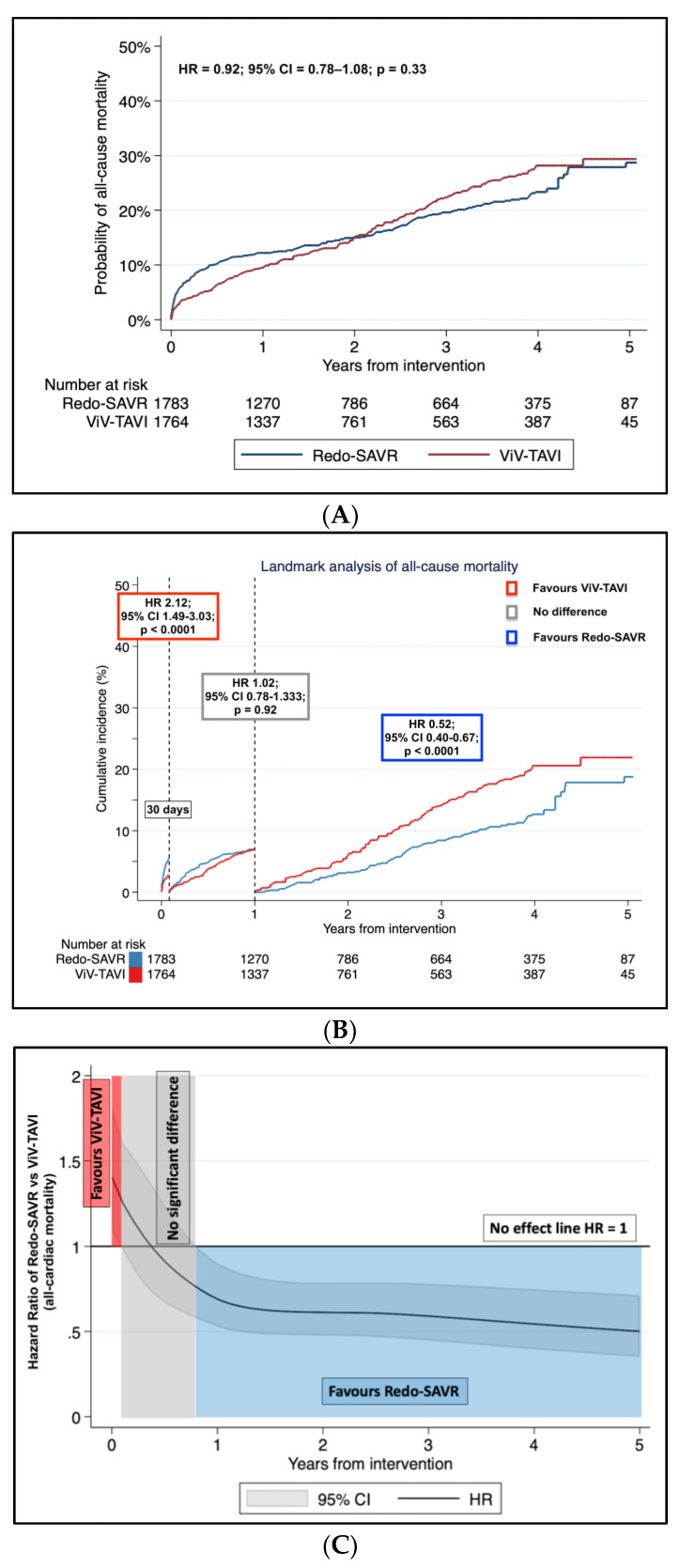
(**A**): Aggregated Kaplan–Meier incidence of all-cause mortality in Redo aortic valve replacement (Redo-SAVR) and valve-in-valve transcatheter aortic valve implantation (ViV-TAVI). (**B**): Landmark analysis of all-cause mortality in Redo-SAVR and ViV-TAVI. Within 30 days from intervention, ViV-TAVI shows a better outcome compared to Redo-SAVR; no difference between the two interventions is reported within 30 days and 12 months; after 12 months, Redo-SAVR shows a better outcome. (**C**): Analysis of the hazard ratio trend over time for all-cause mortality in Redo aortic valve replacement (Redo-SAVR) and valve-in-valve transcatheter aortic valve implantation (ViV-TAVI). The hazard ratio trend was estimated with the fully parametric generalized survival models. The red area shows advantage of ViV-TAVI; the grey area shows no difference between the two interventions; the blue area shows an advantage of Redo-SAVR. The black horizontal line is set at 1 hazard ratio (no effect between the two interventions). HR: hazard ratio; CI: confidence interval.

Figure 2A shows the aggregated Kaplan–Meier failure estimates. The Cox regression linear frailty model revealed no difference between the two groups (HR = 0.92; 95% CI = 0.78–1.08; *p* = 0.33). Incidence of all-cause mortality at one year was 12.9% (95% CI, 11.3%-14.6%) and 9.9% (95% CI, 8.5–11.5%), in Redo-SAVR and ViV-TAVI populations, respectively; incidence of all-cause mortality at five years was 25.4% (95% CI, 22.6–28.6%) and 27.6% (95% CI, 24.9–30.1%), in Redo-SAVR and ViV-TAVI, respectively. The proportional hazard assumption was violated (*p* = 0.005); hence, we performed the landmark analysis and modeled the time-varying hazard ratio. Following the visual inspection of scaled Schoenfeld residuals, the log-log survival, the predicted versus observed survival curves (Supplementary materials, Figures S1–S3) and the Kaplan–Meier curves, we applied the cut-offs of 30 days and 1 year. The selected time points might also have the following rationale: (i) the 30-day cut-off point separates early events, which could be attributed to a clear higher risk of conventional surgery, from late events that are not affected by the intervention procedure period; (ii) the 1-year cut-off point includes patients of all-studies, as studies have at least 1-year follow-up. The landmark analysis showed that Redo-SAVR was associated with a significantly higher incidence of mortality in the first 30 days (HR = 2.12; 95% CI = 1.49–3.03; *p* < 0.0001) whereas no difference was observed between 30 days and 1 year (HR = 1.03; 95% CI = 0.78–1.33; *p* = 0.92). In one year, there was a gradual reversal of the HR, favoring the Redo-SAVR over ViV-TAVI (HR = 0.52; 95% CI = 0.40–0.67; *p* < 0.0001) (Figure 2B). 

The analysis of the HR trend over time of Redo-SAVR versus ViV-TAVI reflected the results of the landmark analysis. (Figure 2C).

### 3.2. Sensitivity Analysis

We performed a sensitivity analysis analyzing the primary endpoint among the six PSM studies included in the meta-analysis. A total of 2340 patients were included in this subgroup, accounting for 1170 patients in each group. 

**Figure 3:**
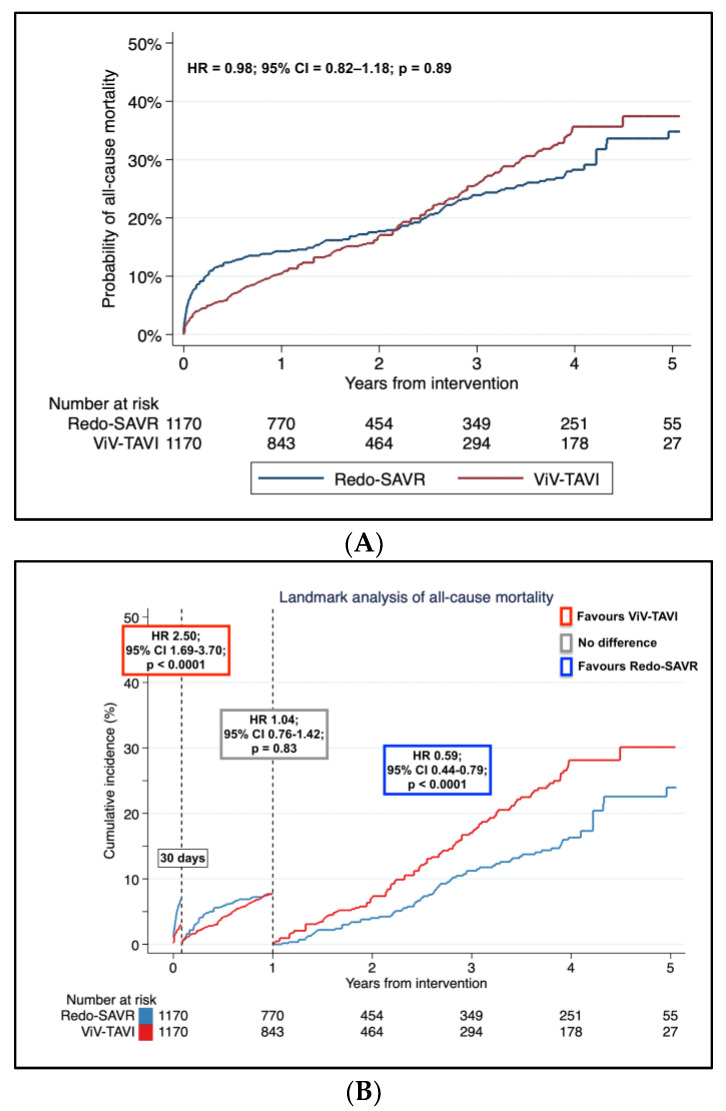

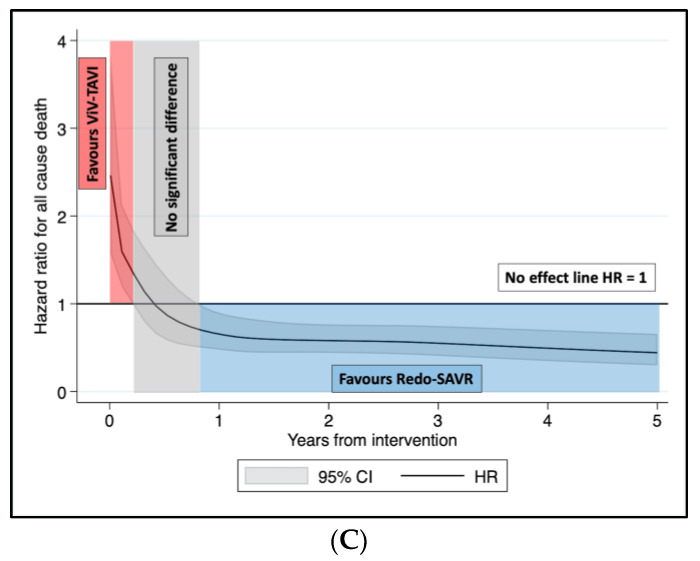
(**A**): Pooled Kaplan–Meier incidence of all-cause mortality of 6 propensity score matched studies in Redo aortic valve replacement (Redo-SAVR) and valve-in-valve transcatheter aortic valve implantation (ViV-TAVI). (**B**): Landmark analysis of all-cause mortality of 6 propensity score matched studies in Redo-SAVR and ViV-TAVI. Within 30 days from intervention, ViV-TAVI shows a better outcome compared to Redo-SAVR; no difference between the two interventions is reported within 30 days and 12 months; after 12 months, Redo-SAVR shows a better outcome. (**C**): Analysis of the hazard ratio trend over time for all-cause mortality of 6 propensity score matched studies in Redo aortic valve replacement (Redo-SAVR) and valve-in-valve transcatheter aortic valve implantation (ViV-TAVI). The hazard ratio trend was estimated with the fully parametric generalized survival models. The red area shows advantage of ViV-TAVI; the grey area shows no difference between the two interventions; the blue area shows an advantage of Redo-SAVR. The black horizontal line is set at 1 hazard ratio (no effect between the two interventions). HR: hazard ratio; CI: confidence interval.

The pooled Kaplan–Meier failure estimates are shown in Figure 3A. The Cox regression linear frailty model revealed no difference between the two groups (HR = 0.98; 95% CI = 0.82–1.18; *p* = 0.89). Incidence of all-cause mortality at one year was 15.2% (95% CI, 13.2–17.6%) and 10.1% (95% CI, 9.1–12.8%), in Redo-SAVR and Viv-TAVI populations, respectively; incidence of all-cause mortality at five years were 31.6% (95% CI, 27.7–35.8%) and 35.4% (95% CI, 31.3–40%), in Redo-SAVR and Viv-TAVI, respectively.

The test for the proportional hazard assumption was violated (*p* < 0.0001). Therefore, following the visual inspection of scaled Schoenfeld residuals, the log–log survival, the predicted versus observed survival curves (Supplementary materials, Figures S4–S6) and the Kaplan–Meier curves, we applied the cut-offs of 30 days and 1 year. The landmark analysis showed that Redo-SAVR was associated with a significantly higher incidence of mortality within 30 days from intervention (HR = 2.50; 95% CI = 1.69–3.70; *p* < 0.0001). No difference between the two interventions was observed from 30 days to 1 year (HR = 1.04; 95% CI = 0.76–1.42; *p* = 0.83), whereas from 1 year to 5 years follow-up, Redo-SAVR showed gradually lower mortality compared to ViV-TAVI (HR = 0.59; 95% CI = 0.44–0.79; *p* < 0.0001) (Figure 3B). 

The analysis of the time-varying HR of Redo-SAVR versus ViV-TAVI was concordant with the results of the landmark analysis (Figure 3C).

### 3.3. Secondary Endpoint: Incidence of Cardiovascular Death

Among the 12 studies included in the meta-analysis, 2 of them [26,29] reported data related to freedom from CV death. A total of 1692 patients were included, 828 subjects in the Red-SAVR groups and 864 patients in the ViV-TAVI group.

**Figure 4:**
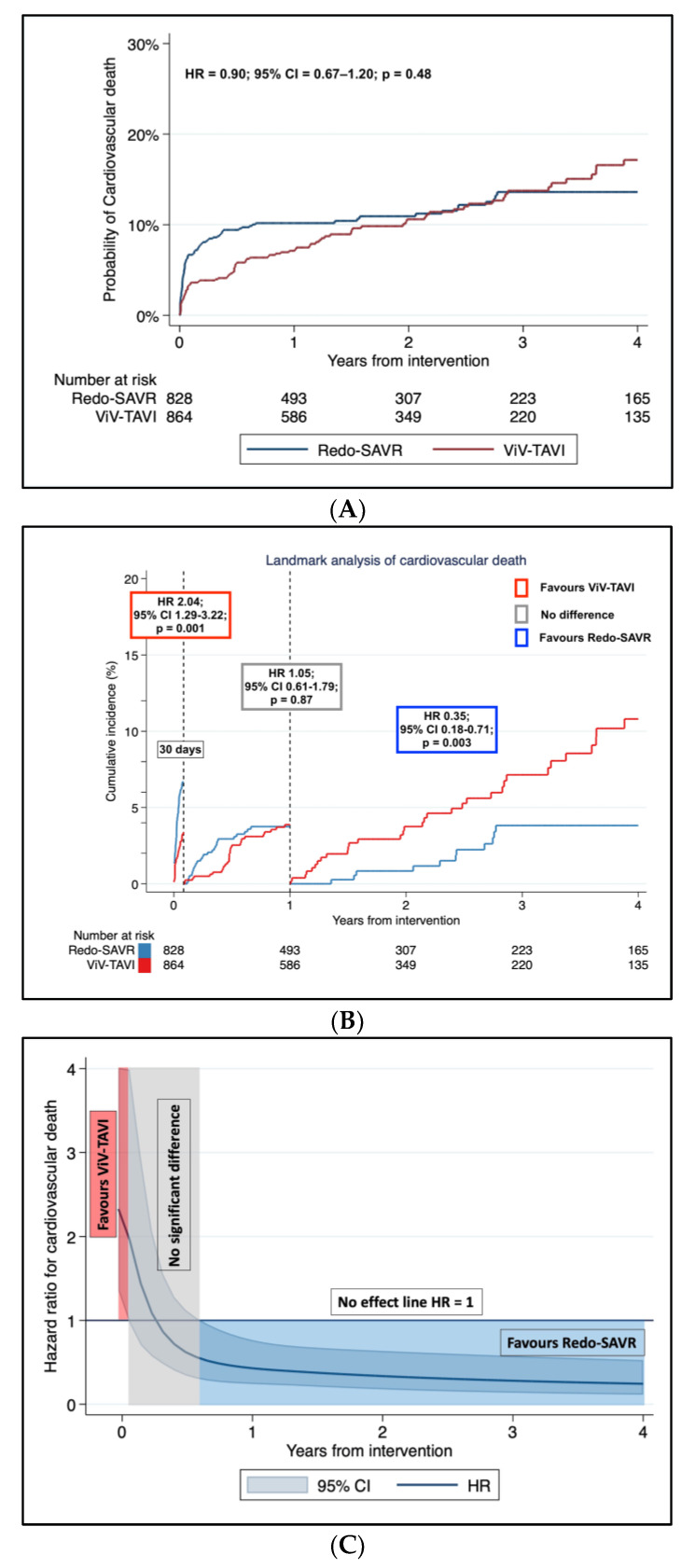
(**A**): Pooled Kaplan–Meier incidence of cardiovascular death in Redo aortic valve replacement (Redo-SAVR) and valve-in-valve transcatheter aortic valve implantation (ViV-TAVI). (**B**): Landmark analysis of cardiovascular death in Redo-SAVR and ViV-TAVI. Within 30 days from intervention, ViV-TAVI shows a better outcome compared to Redo-SAVR; no difference between the two interventions is reported within 30 days and 12 months; after 12 months, Redo-SAVR shows a better outcome. (**C**): Analysis of the hazard ratio trend over time for cardiovascular death in Redo aortic valve replacement (Redo-SAVR) and valve-in-valve transcatheter aortic valve implantation (ViV-TAVI). The hazard ratio trend was estimated with the fully parametric generalized survival models. The red area shows advantage of ViV-TAVI; the grey area shows no difference between the two interventions; the blue area shows an advantage of Redo-SAVR. The black horizontal line is set at 1 hazard ratio (no effect between the two interventions). HR: hazard ratio; CI: confidence interval.

The Cox regression frailty linear model revealed no difference between the two groups (HR = 0.90; 95% CI = 0.67–1.20; *p* = 0.48). The incidence of CV death at one year was 11.6% (95% CI, 9.4–14.1%) and 7.5% (95% CI, 5.9–9.7%) in Redo-SAVR and ViV-TAVI populations, respectively; incidence of CV death at four years was 14.8% (95% CI, 12.1–18.1%) and 17.1% (95% CI, 13.7–21.2%), in Redo-SAVR and Viv-TAVI, respectively. The test for the proportional hazard assumption was violated (*p* < 0.0001). The landmark analysis showed that Redo-SAVR was associated with a significantly higher incidence of CV death (HR = 2.04; 95% CI = 1.29–3.22; *p* = 0.001) within one month from intervention. From one month to one year, there was no difference between the two interventions (HR = 1.05; 95% CI = 0.61–1.79; *p* = 0.87), whereas from one-year up to four-years follow-up, Redo-SAVR showed a reduced incidence of CV death compared to ViV-TAVI (HR = 0.35; 95% CI = 0.18–0.71; *p* = 0.003) (Figure 4A,B).

The analysis of the HR trend over time of Redo-SAVR versus ViV-TAVI was consistent with the results of the landmark analysis (Figure 4C).

## 4. Discussion

TAVI for AS is considered a valid alternative to SAVR, and many authors have reported a non-inferiority of transcatheter therapy to conventional surgery in all surgical risk levels [33,34,35,36]. However, in a recent study-level meta-analysis of seven randomized trials, Barili et al. observed an increased rate of all-cause mortality and composite outcome in TAVI patients compared to SAVR subjects after 24 months of the intervention [37]. Similar outcomes were reported in a large nationwide database (the German Aortic Valve Registry), although TAVi patients received an early-generation bioprosthesis [38]. Conversely, there are no randomized trials comparing long-term results between Redo-SAVR and ViV-TAVI, and Heart Teams base their decisions on data and results of observational retrospective studies.

In this reconstructed individual time-to-event data meta-analysis of 12 studies, we estimated the incidence of all-cause mortality and the incidence of CV death at a five-year follow-up. The main findings of this study are as follows: (i) the incidence of all-cause mortality within one month from the intervention was clearly higher in patients who underwent Redo-SAVR, while this difference was not relevant from one month to one year, and afterward, the incidence of all-cause mortality became in favor of surgery following one year from intervention; (ii) similar trend was found at the sensitivity analysis of 6 PSM studies; and (iii) mortality for CV death was markedly higher within the first 30 days following intervention in Redo-SAVR group and this trend significantly inverted following one year up to four years of follow-up.

Our results confirm the advantages of ViV-TAVI over Redo-SAVR in the very early postoperative follow-up, as already recently described [11,12,13,14]. The higher invasive modalities of the surgical approach compared to the transcatheter one may explain the higher incidence of mortality for any cause and for CV causes. Patients who underwent Redo-SAVR share the non-neglectable negative biological impact of deep anesthesia, re-sternotomy, cardiopulmonary bypass, aortic cross-clamp, blood transfusion requirement, longer intubation time and longer intensive hospital stay. The choice between Redo-SAVR and ViV-TAVI is a very challenging and complex decision-making procedure. The Heart Team plays an important role in this process, as outlined in the latest guidelines [1]. Therefore, a standardized approach and detailed pre-intervention planning are the keys to success. In daily practice, Redo-SAVR is often the first choice for patients with few comorbidities and longer life expectancy, while ViV-TAVI is considered the first option for high-risk and frail patients. However, in the past decade, transcatheter therapies have improved with increased operator experience, perioperative management, and evolving valve design, and these factors are leading ViV-TAVI to become increasingly common in younger patients [39].

Recently, some meta-analyses have reported that ViV-TAVI is associated with lower 30-day mortality, lower incidence of postoperative stroke, lower incidence of major bleeding and less hospital stay, while the mean postoperative valve gradient, the patient-prosthesis mismatch and the paravalvular leak were reported to be markedly lower in Redo-SAVR [11,12,13]. All these studies have reported no inferiority of ViV-TAVI compared to Redo-SAVR in terms of short and long-term mortality. In our study, we tried to analyze the course over time of both interventions, focusing on the time-to-event data reconstruction and on the variation of the HRs over time. Landmark analysis allowed us to define the behavior of the two groups following landmark time cut-off points, which were selected on the basis of Schoenfeld residuals and the visual inspection of Kaplan–Meier curves. Interestingly, the incidence of all-cause mortality after one year was lower in Redo-SAVR, even in the sensitivity analysis of 6 PSM studies. If the higher short-term mortality following Redo-SAVR is easily understandable due to the high surgical risk, much more complex becomes the search for causes that can be associated with a reversal of the mortality trend in the medium and long-term in favor of surgery. One of the conditions linked with higher late mortality after ViV-TAVI is the higher rate of PPM compared to Redo-SAVR. In the Society of Thoracic Surgeons/American College of Cardiology Transcatheter Valve Therapy Registry (TVT Registry) [40], severe PPM was reported in 12% of 62,125 TAVI patients and it was a predictor of one-year mortality (adjusted HR = 1.19, CI 95% 1.09–1.31). TVT Registry also reported a rate of 14.7% of severe PPM in ViV-TAVI. These results are in contrast with the Valve-in-Valve International Data (VIVID) Registry results on 910 patients who underwent ViV-TAVI. The VIVID Registry has observed that post-intervention elevated transvalvular gradient did not affect the 12-month survival [41]. Indeed, the effects of moderate-to-severe PPM on long-term survival were deeply investigated in patients who underwent SAVR, and the impact of PPM on late survival was observed at least after three years following surgery [42]. Valve-in-Valve International Data (VIVID) Registry also reported a significant negative impact on long-term survival and freedom from valve reintervention in those patients who received a ViV-TAVI for a small failed biological, which exhibited frequent PPM [43]. Therefore, it is reasonable to speculate that also in ViV-TAVI, the impact of moderate-to-severe PPM might be observed after three years. Therefore, further studies are mandatory to investigate this relevant issue. The causes of post-implant moderate to severe PPM are multifactorial, and several pre-ViV-TAVI factors were observed, such as a stented surgical bioprosthesis, a small surgical biological valve, a severely stenosed bioprosthesis [41,44] and the intra-annular position of the depth of implant device [43].

Two studies [26,28] included in this meta-analysis reported mid-term data on mortality for CV causes. At landmark analysis, we observed that Redo-SAVR was associated with a higher incidence of CV death within one year from the operation, while this trend reversed in favor of surgery after one year and up to four years of follow-up. These results deserve a word of caution because of the different survival and sample size reported in the two included studies. We can speculate that the better freedom from CV death reported after 1-year in our meta-analysis may be explained by the more frequent incidence of pre-existing surgical valve PPM and valve thrombosis in those patients receiving ViV-TAVI [42]. 

### Limitations

This meta-analysis has several limitations.

First, some studies included in the meta-analysis reported limited follow-up, no more than one year. This may have some implications for the interpretation of the landmark analysis. Second, most studies have a small sample size. Third, randomized studies are lacking, and therefore, only retrospective observational studies were included with a non-neglectable risk of treatment allocation bias and high heterogeneity between the two treatments. Patients who underwent ViV-TAVI have a higher burden of surgical risk factors compared to redo-SAVR, and many of them are even excluded from conventional surgery. The selection criteria for either redo-SAVR or ViV-TAVI indications are different in the included studies, which makes even more relevant the risk for allocation bias. To control for the allocation bias, we performed a sensitivity analysis including only propensity score matching studies, although the bias due to unmeasured variable remains uncontrollable. Fourth, there are lacking data related to outcome depending on the type of degenerated prosthesis (pericardial versus porcine), the type of a newly implanted bioprosthesis valve in redo-SAVR (stented, stentless, rapid deployment), the type of implanted TAVI (self-expanded versus balloon-expandable) and the type of TAVI approach (transapical, transfemoral, trans-axillary). Fifth, only two studies reported cardiovascular mortality. Last, the procedural learning curve, the operator’s skills and expertise, the improvement in material and valve design and the improvement in technical aspects were not investigated since those data were not reported in the included studies. 

## 5. Conclusions

In this meta-analysis with reconstructed time-to-event data of patients who underwent either redo-SAVR or ViV-TAVI for degenerated BP valve, although it seems there is not a relevant difference between the two treatments in the five-year cumulative results, we observed an evident time-varying trend of the HR. ViV-TAVI is associated with lower mortality within the 30 days following the intervention, and this advantage disappeared between 30 days and 1 year and reversed in favor of redo-SAVR 1 year after the intervention. RCTs with longer follow-ups and large multicenter registries are essential to better analyze and define the differences in survival between these two procedures.

## Data Availability

The data presented in this study are available on request from the corresponding author.

## Supplementary Materials

The following supporting information can be downloaded at: https://www.mdpi.com/article/10.3390/jcm12020541/s1, Supplementary PRISMA checklist S1; Table S1: Literature search algorithm; Table S2: Newcasle-Ottawa Scale (NOS) for the risk of bias and quality assessment of Non-Randomized Trials (NRSs); Table S3: Studies characteristics and baseline variables; Table S4: postoperative variables; Figure S1: Scaled Schoenfeld residuals of 12 included studies for all-cause mortality; Figure S2: The log-log survival plot of 12 studies for all-cause mortality; Figure S3: The predicted versus observed survival curves for all-cause mortality of 12 included studies; Figure S4: Scaled Schoenfeld residuals of 6 propensity score matching studies for all-cause mortality; Figure S5: The log-log survival plot of 6 propensity score matching studies for all-cause mortality; Figure S6: The predicted versus observed survival curves of 6 propensity score matching studies for all-cause mortality.
