# Supplementary material for: Redo Surgical Aortic Valve Replacement versus Valve-In-Valve Transcatheter Aortic Valve Implantation: A Systematic Review and Reconstructed Time-To-Event Meta-Analysis
Source: J Clin Med. 2023 Jan 9;12(2):541. doi: 10.3390/jcm12020541 (PMC9866823; doi:10.3390/jcm12020541)
Supplement: Supplementary file 1 [file jcm-12-00541-s001.zip › jcm-2082692-supplementary.pdf]

Supplementary Materials

Redo Surgical Aortic Valve Replacement Versus Valve-In-Valve Transcatheter Aortic Valve Implantation: A Systematic Review and Reconstructed Time-To-Event Meta-Analysis

Francesco Formica [1,*], Alan Gallingani [2], Domenico Tuttolomondo [3], Daniel Hernandez-Vaquero [4], Stefano D'Alessandro [5], Claudia Pattuzzi [1,2], Mevlüt Çelik [6], Gurmeet Singh [7], Evelina Ceccato [1,8], Giampaolo Niccoli [1,3], Roberto Lorusso [9] and Francesco Nicolini [1,2]

[1] Department of Medicine and Surgery, University of Parma, 43124 Parma, Italy
[2] Cardiac Surgery Clinic, University Hospital of Parma, Italy
[3] Cardiology Unit, University Hospital of Parma, Italy
[4] Cardiac Surgery Department, Hospital Universitario Central de Asturias, 33004 Oviedo, Spain
[5] Cardiac Surgery Unit, San Giovanni Bosco Hospital, 10144 Turin, Italy
[6] Department of Cardiothoracic Surgery, Erasmus University Medical Center, 3062 Rotterdam, The Netherlands
[7] Division of Cardiac Surgery, Department of Critical Care Medicine, Mazankowski Alberta Heart Institute, University of Alberta, Edmonton, AB 11220, Canada
[8] Medical Library, University of Parma, 43124 Parma, Italy
[9] Cardiovascular Research Institute Maastricht (CARIM), Maastricht, P.O. Box 616, Maastricht, The Netherlands
* Correspondence: francescoformica.hs@gmail.com (F.F.)

Table S1. Search algorythm.

PUBMED		
**Query**	**Search Details**	**Results**
**#1**	("redo surgical aortic valve replacement"[Title/Abstract] OR "Redo-SAVR"[Title/Abstract] OR "reoperation"[Title/Abstract] OR "reoperative"[Title/Abstract]) AND (2000/1/1:2022/09/30[pdat]) 2022/09/30	31,657
**#2**	"ViV-TAVI"[Title/Abstract] OR "ViV-TAVR"[Title/Abstract] OR "valve-in-valve"[Title/Abstract]) AND (2000/1/1:2022/09/30[pdat]) 2022/09/30	1,653
**#3**	**#1 AND #2**	**207**
**EMBASE**		
**#1**	('redo surgical aortic valve replacement' OR 'redo savr' OR 'reoperation' OR reoperative) AND [2000-2022]/py AND [01-01-2000]/sd NOT [01-06-2022]/sd	94,572
**#2**	('viv-tavi' OR 'viv-tavr' OR 'valve-in-valve') AND [2000-2022]/py	2,693
**#3**	**#1 AND #2**	**490**
**CENTRAL**		

**#1**	redo surgical aortic valve replacement:ti OR Redo-SAVR:ti,ab,kw OR reoperation:ti,ab,kw OR reoperative:ti,ab,kw with Cochrane Library publication date Between Jan 2000 and May 2022, in Trials	4885
**#2**	(ViV-TAVI):ti,ab,kw OR (ViV-TAVR):ti,ab,kw OR (Valve-in-vave):ti,ab,kw OR ("transcatheter"):ti,ab,kw with Cochrane Library publication date Between Jan 2000 and Sep 2022, in Trials	2215
**#3**	**#1 AND #2**	**28**

Table S2. NOS for the risk of bias and quality assessment of NRSs

Author	Year	Selection				Comparability	Exposure			Total score
		Adequate definition of patient cases	Representativeness of patient cases	Selection of controls	Definition of controls	Control for important or additional factors	Ascertainment of exposure	Same method of ascertainment for participants	Nonresponse rate	
Deharo et al.	2020	⋆	⋆	⋆	⋆	⋆	⋆	⋆	⋆	8
Dokollari et al.	2021	⋆	⋆	⋆	⋆	⋆	⋆	⋆	⋆	8
Ejiofor et al.	2016	⋆	⋆	⋆	⋆	⋆⋆	⋆	⋆	⋆	9
Patel et al.	2021	⋆	⋆	⋆	⋆	⋆	⋆	⋆	⋆	8
Seedek et al.	2019	⋆	⋆	⋆	⋆	⋆	⋆	⋆	⋆	8
Silaschi et al.	2017	⋆	⋆	⋆	⋆	⋆	⋆	⋆	⋆	8
Maslow et al. [21]	2010	⋆	⋆	⋆	⋆	⋆	⋆	⋆	⋆	8
Stankoski et al.	2020	⋆	⋆	⋆	⋆	⋆	⋆	⋆	⋆	8
Tam et al.	2020	⋆	⋆	⋆	⋆	⋆⋆	⋆	⋆	⋆	9
Woitek et al.	2020	⋆	⋆	⋆	⋆	⋆	⋆	⋆	⋆	8
van Steenbergen et al.	2011	⋆	⋆	⋆	⋆	⋆	⋆	⋆	⋆	8
Hernandez-Vaquero et al.	2019	⋆	⋆	⋆	⋆	⋆⋆	⋆	⋆	⋆	9
Spaziano et al.	2017	⋆	⋆	⋆	⋆	⋆	⋆	⋆	⋆	8

Table S3. Studies characteristics and baseline variables.

Study (1st Author; Year)	Ejiofor 2016	Silaschi 2016	Spaziano 2017	Seedek 2019	Hernandez -Vaquero 2019	Deharo 2020	Stankowski 2020	Tam 2020	Woitek 2020	Dokollari 2021	Patel 2021	Steenbergen 2021
**Country**	USA; Boston	Uk, Germany	Canada, Ireland, Denmark, France, Italy and Germany	USA, Rochester	Spain, Oviedo	France, Marselle	Germany, Poland	Canada, Toronto	Germany, Leizig	Canada, Toronto	USA, Atlanta	The Netherland
**Study Design**	PSM/ Retrospective	Retrospective	PSM/Retrospective	Retrospective	PSM/Retrospective	PSM/ Retrospective	Retrospective	PSM/ Retrospective	Retrospective	Retrospective	Retrospective	PSM/ Retrospective
**Study period**	2002-2015	2008-2015	2007-2015	2008-2018	2012-2018	2010-2019	2003-2018	2008-2017	2006-2017	2010-2018	2012-2019	2014-2018
**Redo-SAVR total patients**	22	59	78	260	57	717	40	131	111	31	86	165
**ViV-TAVI total patients**	22	71	78	90	57	717	68	131	147	57	187	165
**Male gender,(%)**												

**All studies**	61,3	59,2	53	71	43	56,9	49	61,8	61,2	50	67,3	59,4
**Redo-SAVR**	59,1	61	56,5	68	38	57,7	62,5	61,8	59,9	54,8	66,3	60,6
**ViV-TAVI**	14	57,7	50	81	50,8	56,1	41,2	61,8	62,6	50,9	67,9	58,2
**Age (mean)**												
**All studies**	74,8			73	79,3	74,7		76			67,1	73,6
**Redo-SAVR**	74,5	72,9	77,4	71	78,8	74,5	72,9	76,1	58,5	67,2	61,3	73
**ViV-TAVI**	75	78,6	78	79,2	79,8	74,9	79,2	75,9	76,2	79,06	73	74,2
**Previous AMI,(%)**												
**All studies**			37			14,8			7,7	3,4	23,4	
**Redo-SAVR**			32			14,9			6,3	3,5	12,8	
**ViV-TAVI**			42			14,6			8,8	3,2	28,3	
**Previous PCI,(%)**												
**All studies**						13,9		9,5	12,8			
**Redo-SAVR**						13,5		11,5	6,3			
**ViV-TAVI**						14,4		7,6	17,7			
**Previous CABG,(%)**												
**All studies**	59,1	25,4	27	34		23,5	31,5	37,4	20,5	22,7	38,8	
**Redo-SAVR**	54,4	16.9	23	29		22,3	20	30,5	9,9	17,5	22,1	
**ViV-TAVI**	63,6	32,4	31	48		24,8	38,2	44,3	32,7	32,3	46,5	
**EF (mean)**												
**All studies**	55			59,5							51	52,1
**Redo-SAVR**	55		49,5	61,2	53				57,4	50,46	53,4	52
**ViV-TAVI**	55	n,a,	50,7	54,7	57,7				54,5	49	48,7	52,3
**AF,(%)**												
**All studies**			36	37		61	45,3	40,4	33,7	32,9	38,4	
**Redo-SAVR**			37	33		60,8	40	38,9	18,9	29,8	24,7	

**ViV-TAVI**			35	47		61,2	48,5	42	44,2	38,7	44,9	
**Hypertension,(%)**												
**All studies**	93,2		72,5	77		78,5	91,6	90	93	85,2	90,4	
**Redo-SAVR**	90,9		73	73		77,8	90	88.5	86,5	82,5	83,7	
**ViV-TAVI**	95,5		72	88		79,4	92,6	91,6	98	90,3	93,6	
**Diabetes (%)**												
**All studies**	34,1	10,8	17	23		31	37,9	48,4	27,5	26,1	37,7	19
**Redo-SAVR**	22,7	10,2	15	22		30,3	45	47,3	16,2	28,1	34,9	17,6
**ViV-TAVI**	45,5	11,3	19	28		31,7	33,8	49,6	36,1	22,6	39	20,6
**Previous Stroke,(%)**												
**All studies**	18,2	12,3	10			5	8,3	12,5	8,1	26,1	27,8	9,7
**Redo-SAVR**	13,6	10,2	12			5	5	12,2	7,2	31,6	26,7	10,3
**ViV-TAVI**	22,7	14,1	9			5,3	10,3	13	8,8	16,1	28,3	9,1
**Renal failure,(%)**												
**All studies**	22,7					15,5	40,7	9,5	17,4		5,1	
**Redo-SAVR**	18,2					15,2	30	8,4	7,2		3,5	
**ViV-TAVI**	27,3					15,9	47,1	10,7	25,2		5,9	
**Dialysis,(%)**												
**All studies**				2			1,85	14,5	0,8			
**Redo-SAVR**				3			0	14,5	0			
**ViV-TAVI**				1			2,9	14,5	1,4			
**COPD,(%)**												
**All patients**			9,6	7	7	16	17,6	30,1	32,5	17	40,3	16,7
**Redo-SAVR**			8	3	1,8	15,9	20	28,2	10,8	17,5	33,7	12,7
**ViV-TAVI**			12	18	12,3	16,3	16,2	32,1	49,7	16,1	43,3	17

**Peripheral disease,(%)**												
**All studies**	25	23,8	15	24	2,6	36,9	12	8,8	12,4	43,2	22,3	
**Redo-SAVR**	22,7	13,6	17	14	0	36,7	5	9,2	5,4	38,6	9,3	
**ViV-TAVI**	27,3	32,4	14	53	5,3	37,1	16,1	8,4	17,7	51,6	28,3	
**Smoker,(%)**												
**All studies**						14,5	7,4	7,2		34,1		
**Redo-SAVR**						15,2	5	7,6		40,4		
**ViV-TAVI**						13,8	8,8	6,9		22,6		
**Dyslipidemia,(%)**												
**All studies**						53,4	71,3	71,3		78,4		
**Redo-SAVR**						52,9	65	69,5		73,7		
**ViV-TAVI**						54,1	75	73,3		87,1		

Table S4. Postoperative variables

Study (1st Author + Year + Country)	Ejiofor 2016	Silaschi 2016	Speziano 2017	Seedek 2019	Hernandez-Vaquero 2019	Deharo 2020	Stankowski 2020	Tam 2020	Woitek 2020	Dokollari 2021	Patel 2021	van Steenbergen 2021
All-cause death (%), all studies	2,3	4,6	7,4	3	5,2	5,4	5,1	6,25	4,6	4,5	1,4	4,5
Redo-SAVR	4,5	5,1	7,5	3	3,5	7,3	6,4	7,9	4,1	7	1,2	3
ViV-TAVI	0	4,2	7,4	2	7	3,6	3,9	4,2	4,5	0	1,6	6,1
CV-death (%), all studies		3.8				4.7						
Redo-SAVR		5.1				6.6						
ViV-TAVI		2.8				2.9						
AF (%), all studies	40,9			25		2,3	16,6			34,1	11,7	
Redo-SAVR	63,6			28		4	29,2			47,4	22,1	
ViV-TAVI	18,2			13		0,6	5,7			9,7	7	
AKI (%), all studies	6,8	7,7		17					10,5	13,6		
Redo-SAVR	4,5	13,6		16					17,1	15,8		
ViV-TAVI	9,1	2,8		4					5,4	9,7		
Dialysis (%), all studies		4,6	3,7	3			8				1,8	
Redo-SAVR (%)		6,8	7,5	4			12				3,5	
ViV-TAVI, (%)		2,8	1,5	0			4				1,1	
PM implant (%), all studies	4,5	17,7	5,5	16		12	10		13,2	6,8	2,9	4,9
Redo-SAVR, (%)	4,5	25,4	5	19		5,7	10		12,6	8,8	5,8	4,2
ViV-TAVI, (%)	4,5	9,9	5,9	7		18,4	10		13,6	3,22	1,6	5,5

**Stroke (%), all studies**	4,5	1,5	5,5	1		0,7	0,6		6,6	5,7	2,9	1,8
**Redo-SAVR**	9,1	3,4	7,5	1		0,4	0		8,1	7	7	2,4
**Stroke ViV-TAVI**	0	0	4,4	1		1	1		5,4	3,2	1,1	1,2
**Prolonged ventilation (%), all studies**				12								
**Redo-SAVR**				15								
**ViV-TAVI**				3								
**AMI (%), all studies**	0	1,5	1,8			0,28	0,6		2,7		1	
**Redo-SAVR**	0	1,7	2,5			0,4	0		1,8		2,3	
**ViV-TAVI**	0	1,4	1,5			0,1	1		3,4		0,5	
**Major Bleeding (%), all studies**		20,8	12,8	12		4,4			12,8		1,8	
**Redo-SAVR**		33,9	21,6	16		4,7			21,6		2,3	
**ViV-TAVI**		9,9	6,1	1		4			6,1		1,6	
**Re-thoracotomy (%), all studies**	0	8,5		4						10,2	0,7	
**Redo-SAVR**	0	15,3		5						15,8	1,1	
**ViV-TAVI**	0	2,8		1						0	0,5	
**Blood Transfusions (%), all studies**			66,6	43				49,6			33,3	
**Redo-SAVR**			65	53				81,7			72,1	
**ViV-TAVI**			10,3	12				17,6			15,5	
**Peripheral Complication (%), all studies**		13,8					3,7		13,2		0	3,3
**Redo-SAVR**		5,1					0		15,3		0	
**ViV-TAVI**		21,2					5,9		11,6		0	6,7
**PVL (%), all studies**	11,4	19,2	23,1						27,1	15,9	9,5	
**Redo-SAVR**	0	13,6	2,5						19,2	0	2,9	
**ViV-TAVI**	22,7	23,9	35,3						35,2	24,5	16	
**Mean Grad (mmHg), all studies**	12,9										15,4	

**Redo-SAVR**	13,5	12,2	19	15			14,3		11,9	16,2	14,3	
**ViV-TAVI**	12,4	19,7	16,8	21,5			18,1		17,4	16,8	16,6	
**Combined procedure (%), Redo-SAVR**									50.4	40.3	2.1	
**Mean days LOS, all studies**	7,8										4,5	
**Redo-SAVR**	10,5		14				11,4	13,2	11,2	10,2	7	
**ViV-TAVI**	5		9,5				7,1	7,25	8,7	3,6	2	

SAVR, surgical aortic valve replacement; ViV-TAVI, valve-in-valve transcatheter aortic valve implantation; AF, atrial fibrillation; AKI, acute kidney injury; PM, pace-maker; AMI, acute myocardial infarction; PVL, prosthetic valve leakage, LOS, length of stay.

Figure S1. Scaled Schoenfeld residuals of 12 included studies for all-cause mortality

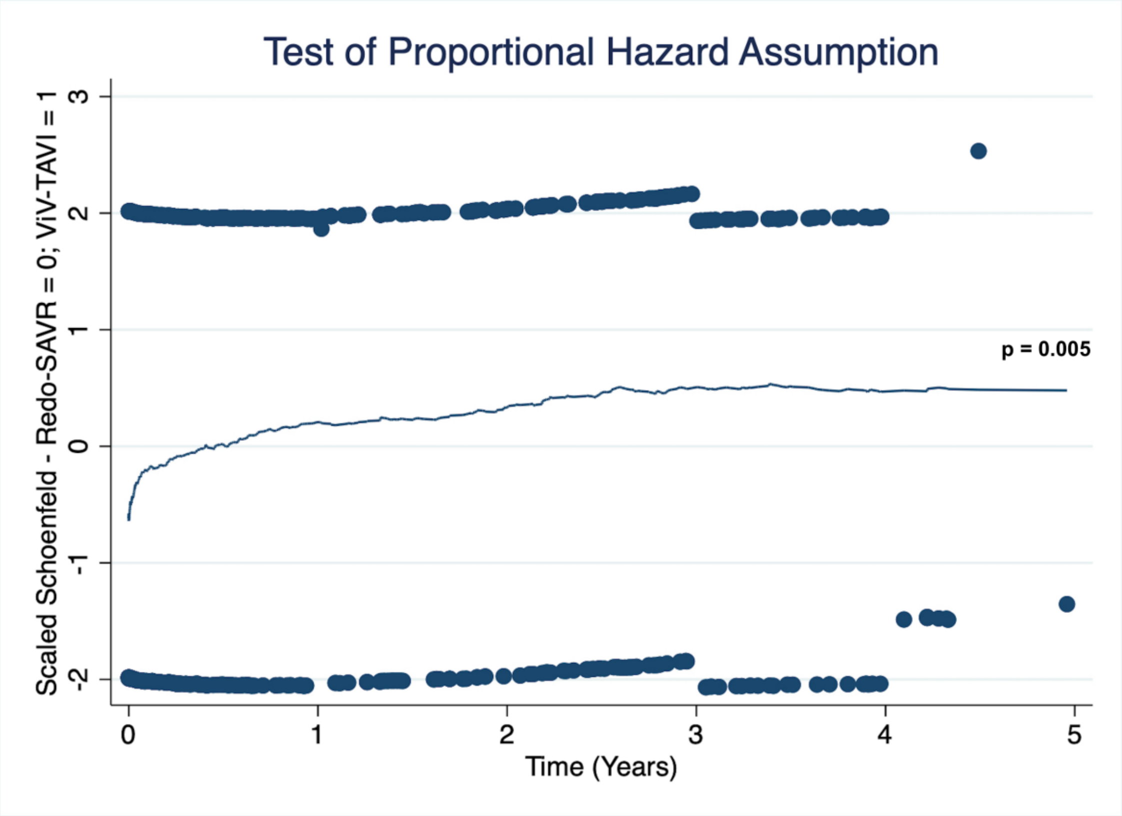

Figure S2. The log-log survival plot of 12 studies for all-cause mortality.

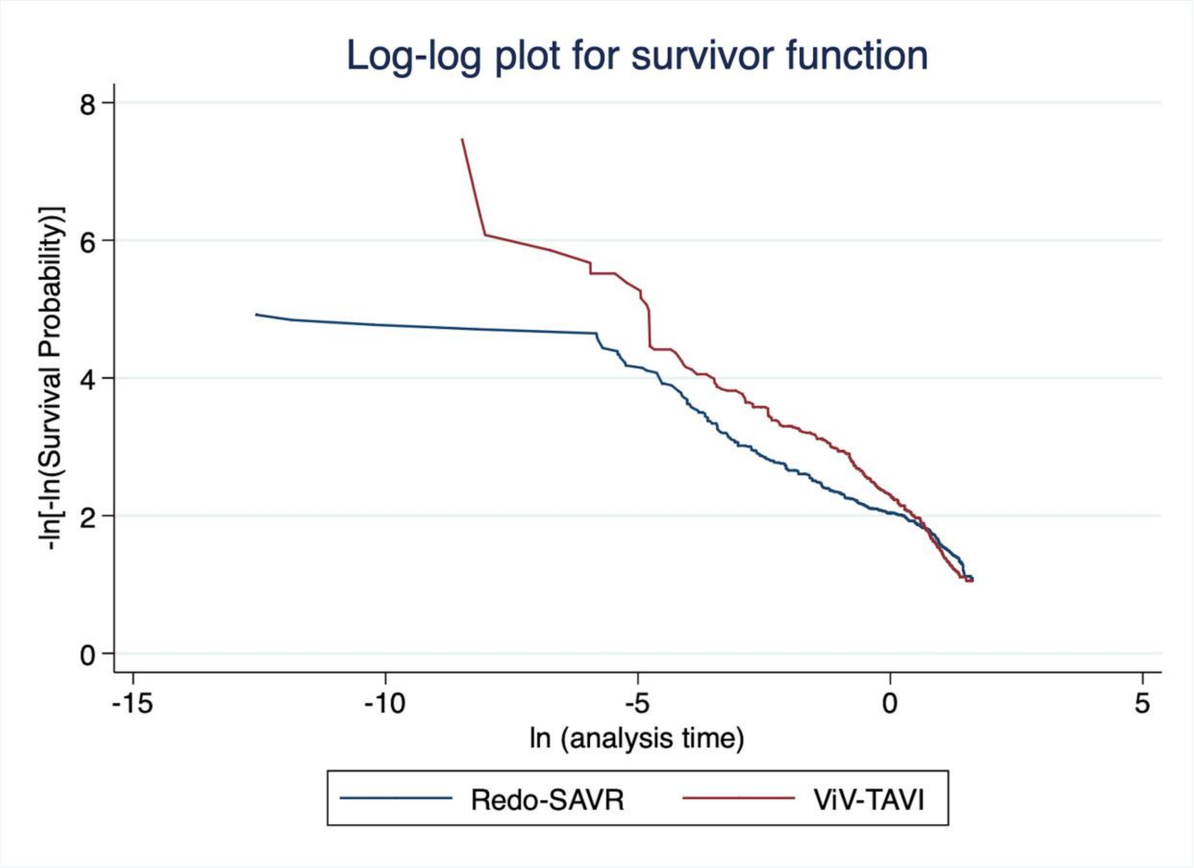

Figure S3. The predicted versus observed survival curves for all-cause mortality of 12 included studies

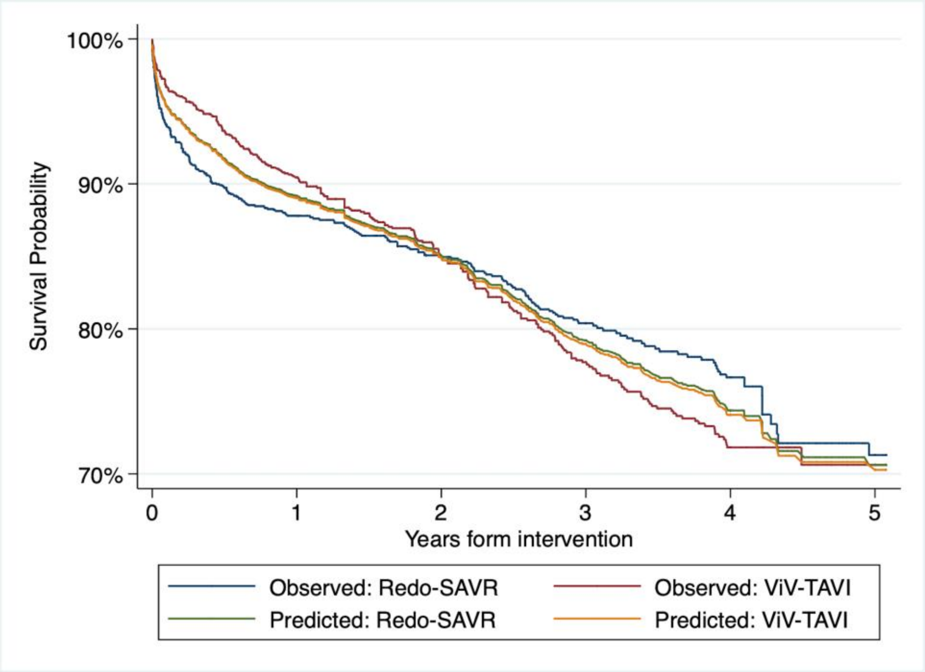

Figure S4. Scaled Schoenfeld residuals of 6 propensity score matching studies for all-cause mortality

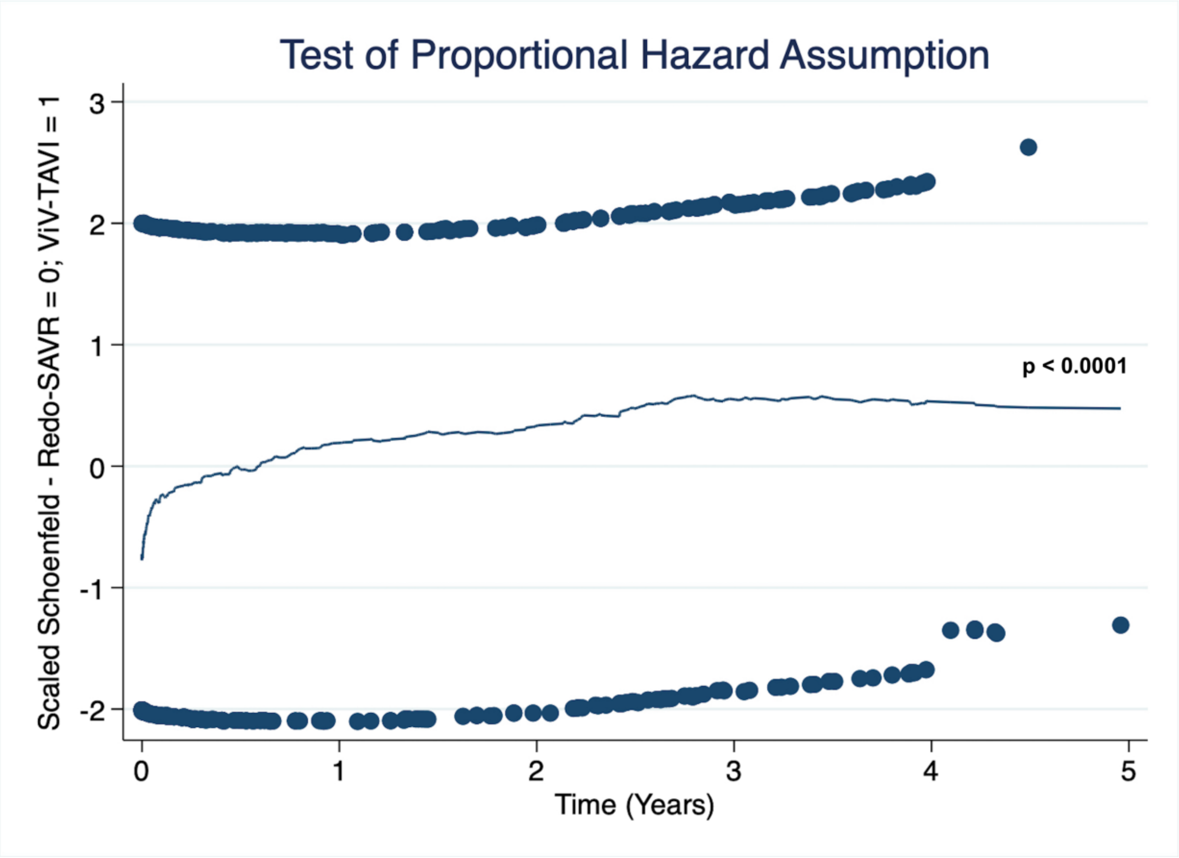

Figure S5. The log-log survival plot of 6 propensity score matching studies for all-cause mortality

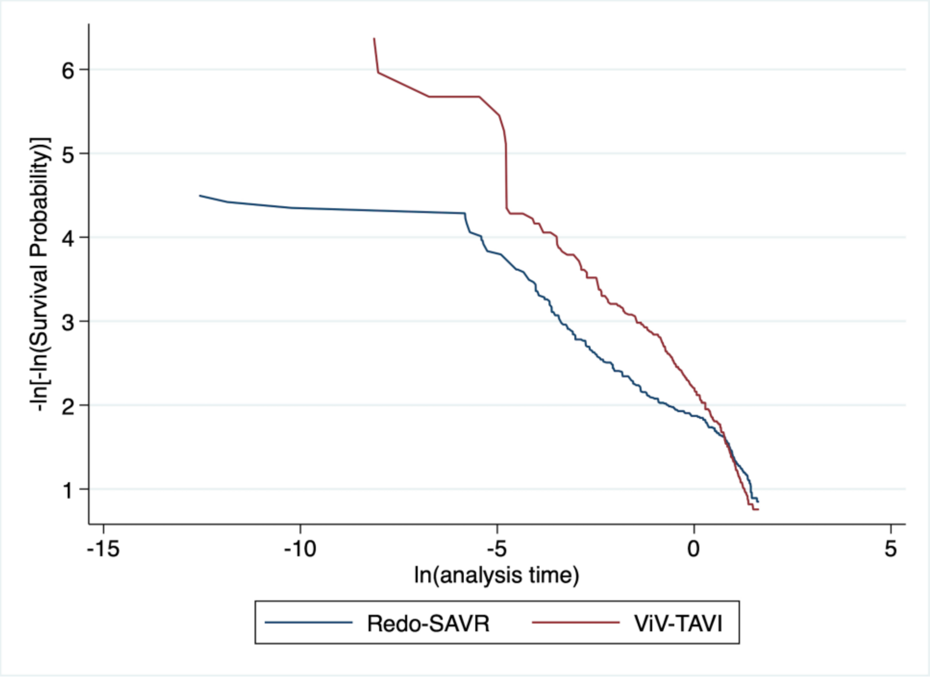

Figure S6. The predicted versus observed survival curves of 6 propensity score matching studies for all-cause mortality

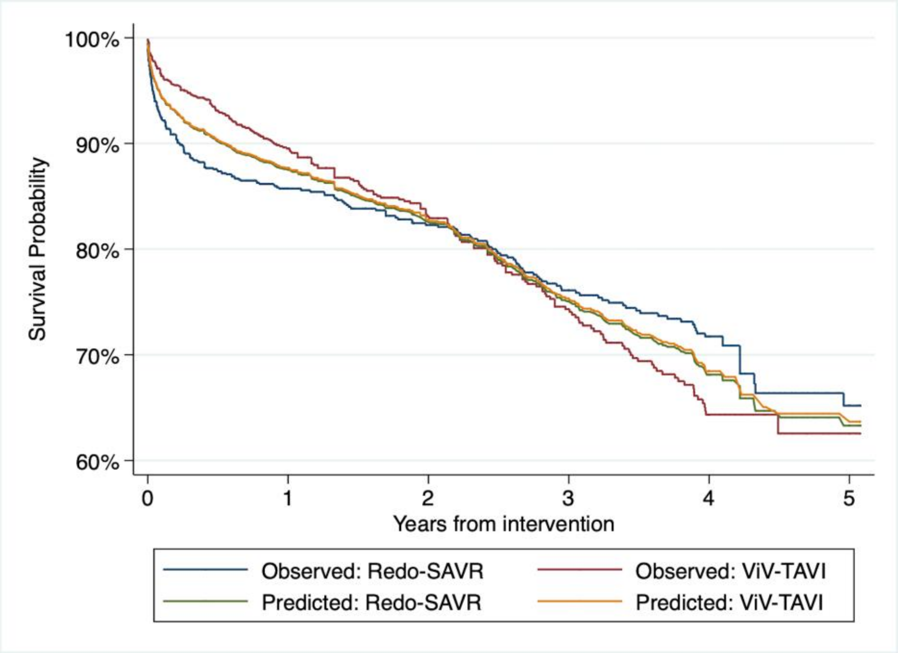

100%
90%
80%
70%
60%
Survival Probability
0
1
2
3
4
5
Years from intervention
Observed: Redo-SAVR
Observed: ViV-TAVI
Predicted: Redo-SAVR
Predicted: ViV-TAVI
